# Impact of Arterial Hypertension on Left Atrial Size and Function

**DOI:** 10.1155/2020/2587530

**Published:** 2020-09-14

**Authors:** Youssef Ikejder, Majda Sebbani, Iliass Hendy, Mounia Khramz, Ali Khatouri, Laila Bendriss

**Affiliations:** ^1^Cardiology Department, Military Hospital in Marrakesh, Morocco; ^2^Community Medicine and Public Health Department, Research Laboratory, Biosciences and Health, School of Medicine, Cadi Ayyad University/ Clinical Research Unit, Mohammed VI University Hospital, Marrakech, Morocco

## Abstract

An increase in left atrial (LA) size in patients with arterial hypertension (AHT) has long been known to be associated with worse cardiovascular morbidity and mortality contributes to various complications, including atrial arrhythmias, stroke, and heart failure. The aim of our study was to evaluate the impact of arterial hypertension (AHT) on the LA size and function. This cross-sectional investigation included one hundred patients with essential hypertension without valvular or structural heart disease and atrial fibrillation. All recruits had a transthoracic echocardiography. LA volumes were measured by area-length method in transthoracic echocardiography at different cardiac cycle times. The indices of LA function were calculated: the reservoir function (total emptying fraction, total emptying volume, and expansion index), the conduit function (passive emptying fraction and passive emptying volume), and the pump function (active emptying fraction and active emptying volume). For all statistical tests, a *p* value ≤0.05 (represents the degree of significance) is considered statistically significant. In univariate analysis, LA was dilated in 9% of patients. The LA reservoir function and the pump function were increased, respectively, in 85% and 82% of patients. LA conduit function was impaired in 80% of patients. In bivariate analysis, the most powerful factors for this repercussion were diabetes (LA volume MAX dilated in nondiabetic patients (*p* = 0.037)), obesity (the reservoir function was impaired in obese patients (*p* = 0.015)), and antihypertensive drugs (the reservoir function was impaired in patients who take beta blockers (*p* = 0.023); the LA pump function was significantly impaired in patients treated with calcium antagonists (*p* = 0.012)). This study proved the impact of AHT on the LA size and function. Further investigations are necessary to evaluate the potential predictive value of LA remodeling in hypertensive patients like speckle tracking imaging.

## 1. Introduction

The left atrial (LA) enlargement is a very important risk factor of cardiovascular mortality and events [1, 2]. The LA phasic function has long been considered unimportant. The number of studies that are demonstrated that the reservoir and the active pump function of LA is increasing [2]. Most researchers agree on the negative impact of hypertension [3–6]. However, some studies do not fully corroborate this result [7–9], this impact can be explained by the left ventricular (LV) remodeling in these patients [10]. Frank-Starling is also valid in the human heart and depends on the systolic and diastolic LV properties that can be changed under many pathophysiological conditions, arterial hypertension, and changes in LV overload [11, 12]. The enlargement of the LA size and function is an important compensatory behavior for changes in critics of the average pressure of the LA or the increased preload; in addition, the increases in LA size and function are parallel to the degree of LV dysfunction [13]. The hypothesis is that AHT influences the LA size and function. The purpose of the present study was to evaluate LA size and phasic function using two-dimensional volumetric parameters in subjects with essential hypertension to establish the prognostic elements of arterial hypertension.

## 2. Methodology

### 2.1. Study Population

This cross-sectional study included 100 patients (58 men and 42 women) referred to cardiology consultation for essential hypertension at the military hospital in Marrakesh. Hypertension was diagnosed as a blood pressure of 140/90 mmHg or as the current use of antihypertensive drugs. We included in this study all patients whose age is more than 18 years and who received at least monotherapy at least one year and for whom transthoracic echocardiography was performed. Patients were excluded if they had atrial fibrillation, significant valvular or structural heart disease, or poor imaging quality. The data is collected from an exploitation sheet. The echocardiographic parameters were taken by the same doctor independently of the clinical data. The statistical analysis is carried out by another doctor in the epidemiology and biostatistics department. Anthropometric measures (height and weight) and laboratory analyses (levels of fasting glucose, total cholesterol, triglycerides, and serum creatinine) were measured in all participants. Body mass index (BMI) was calculated for each patient.

### 2.2. Echocardiography

Echocardiographic examination was performed with a Vivid S6 ultrasound machine. Values of all two-dimensional echocardiographic parameters were calculated as the average value of three consecutive cardiac cycles according to the latest ESC recommendations. The images were recorded for each of the measured parameters. All the recordings were taken during the spatial and temporal resolution (gain, compressions, sweep speed at 100 mm/s, optimum Doppler beam alignment, speed adjustment). The two-dimensional measurements of LA were taken on images centered on it with a spatial resolution. LA anteroposterior diameter: by parasternal *M*-mode long axis view (*N* < 40 mm), LA area in section 4-chamber (*N* < 20 cm^2^). The LA volumes were measured by the Simpson biplane method at different times of the cardiac cycle: end-systole (LA maximum volume (LAVmax) (16 < *N* < 28 ml/m^2^)), at the time of closing mitral (LA minimum volume (LAVmin) (7 < *N* < 15 ml/m^2^)), and immediately before LA contraction (LA pre-A volume (LAVpre-A) (10 < *N* < 20 ml/m^2^)). We calculate also the indices of the reservoir function (total emptying volume (LAVmax – LAVmin)), total emptying fraction (((LAVmax – LAVmin/LAVmax) (*N* > 40%)), the expansion index (no reference value)), the conduit function (passive emptying volume ((LAVmax – LAVpre-A) (*N* ≥ 15 ml)), the passive emptying fraction ((LAVmax – LAVpre-A)/LAVmax) (*N* ≥ 35%)), the pump function (active emptying volume (LAVpre-A – LAVmin), and the active emptying fraction ((LAVpre-A – LAVmin/LAVpre-A) (*N* ≥ 25%)). We did not collect data of the speckle tracking echocardiography for LA phasic function, because we did not have universal and exact reference values for comparison. We took as reference values those of the European Association of Cardiovascular Imaging used also in other studies. LA was considered dilated when LAVmax ≥34 ml/m^2^ associated or not with the increase of other volumetric parameters. The LA reservoir function is impaired when the total emptying fraction is less than 40%. The conduit function is impaired when LA passive emptying fraction is strictly less than 35%± passive emptying volume <15 ml. The pump function is impaired when LA active emptying fraction is strictly less than 25%. LV *M*-mode cut plane on parasternal long axis view: diastolic and systolic thickness of the interventricular septum and posterior wall, left ventricular end-diastolic and end-systolic diameter, and LV mass indexed to the body surface. The end-diastolic and end-systolic LV volumes were measured in 4-chamber and 2-chamber sections for assessment of left ventricular ejection fraction (LVEF) with the Simpson's biplane method. Transmitral Doppler inflow and tissue pulsed Doppler parameters were obtained in the apical 4-chamber view. Pulsed Doppler measurements included transmitral early and late diastolic peak flow velocity (E and A, respectively). Tissue Doppler imaging was used to obtain LV myocardial velocities in the apical 4-chamber view, with a sample volume placed at the septal and lateral segment of the mitral annulus during early diastole (e′). The average of the peak early diastolic relaxation velocity (e′) of the septal and lateral mitral annulus obtained by the tissue Doppler was computed, and the E/e′ ratio was calculated.

### 2.3. Statistical Analysis

Statistical analysis was performed with SPSS V16. Quantitative variables were expressed as numbers, mean ± standard deviation, and compared using a Student's test. Qualitative variables were expressed in percentages, and using a Levene's test and Test's test. Univariate and bivariate analyses are done with chi-square tests, Fisher's exact test, and the Mann–Whitney *U* test. The results were reported as a relative risk with a 95% interval confidence. For all statistical tests, a *p* value ≤0.05 (represents the degree of significance) is considered statistically significant. The bivariate logistic regression analysis included grades of hypertension, age, sex, cardiovascular risk factors, E/e, and LVMI. Heart rate analysis was not included in this study, because the heart rate was normal in most of patients. Some results are noted “not applicable” when the comparison of the percentages was not possible due to the impossibility of verifying the conditions of application of the statistical chi-square test (when the theoretical numbers were less than 5). The intraobserver variability of the LA phasic function was not the subject of the study. The cost of doing and remaking in the same patient makes it difficult to achieve in our context.

## 3. Results

### 3.1. Clinical and Demographic Characteristics

Clinical and demographic characteristics of the population are given in Table 1. The mean age of the subjects was 57.3 years with extremes from 41 years to 79 years. Fifty-eight patients were men with a sex ratio estimated at 1.38. 46 patients were obese (46%), 44 patients were diabetic (44%), and 43 were sedentary (43%). The average of the heart rate was 63,2 bpm. The average of systolic blood pressure was 12,72 mmHg. The average of diastolic blood pressure was 78,3 mmHg. Majority of patients were asymptomatic. The AHT duration was variable. Half of patients had a duration between 1 and 4 years (49%). More than half had AHT grade II (62%). Antihypertensive drugs, calcium antagonists, followed by antagonist converting inhibitors, and ARAII were the most prescribed, respectively, 20%, 39%, and 34%: alone or in combination. Among the 100 patients, 47% were on monotherapy, 40% on dual therapy, and 13% on triple therapy. The prescription was largely dominated by monotherapy.

### 3.2. Left Ventricular Parameters on Echography

They were presented by the following: the end-diastolic dimension (LVEDD) has an average of 49 mm (30 mm-61 mm), and it was increased in 7% of patients; the LV end-systolic dimension (LVESD) has an average of 30.2 mm (11 mm-42 mm), and it was increased in 5% of patients. The left ventricular ejection fraction (LVEF) has an average of 65% (53%-77%), and it was preserved in all patients. The LV mass has an average of 116.7 ± 19 g/m^2^ (82.13 g/m^2^-171.43 g/m^2^), and it was increased in 65% of patients. Of these patients, 20% presented with concentric left ventricular hypertrophy (LVH), and 45% had eccentric LVH; 56% of patients had normal filling pressure with diastolic dysfunction grade I, while 29% had diastolic dysfunction grade II, and 1% had an elevated filling pressure with diastolic dysfunction grade III. For the rest (14%), the diastolic dysfunction was indeterminate (Table 2).

### 3.3. Left Atrial Volumes

The average of LA maximum volume indexed to body surface was 29 ml/m^2^ with a minimum of 12.3 ml/m^2^ and a maximum of 47.30 ml/m^2^, and it was elevated in 09% of patients. The average of LA minimum volume was 11 ml/m^2^, with a minimum of 4.8 ml/m^2^ and a maximum of 25.5 ml/m^2^, and it was elevated in 58%. The LA pre-A volume had an average of 18.5 ml/m^2^ with a minimum of 8.10 ml/m^2^ and a maximum of 32.2 ml/m^2^, and it was increased in 72%. All three volumes were elevated simultaneously in 48% of patients. The maximum volume was increased in 4% of patients, the minimal volume was increased in 6%, and the pre-A volume was increased in 8%. The 2 volumes Vmax-VpréA were increased in association in 15% of patients (Table 2).

### 3.4. Left Atrial Function

The average of total emptying fraction was 54.9% (15%-75%). It was increased in 85% of patients. The average of passive emptying fraction was 24.6% (7%-53%). It was impaired in 80% of patients. The average of active emptying fraction was 39.9% with a minimum was 11% and a maximum was 71%. It was increased in 82% of patients. The average of expansion index was 1.44 (0.54-3.11) (Table 2).

### 3.5. Statistical Analysis

#### 3.5.1. Univariate Analysis

The results of univariate analysis of the variation of LA size and function in the hypertensive patients. LA was dilated in 09% patients. The LA reservoir function and the pump function were increased, respectively, in 85% and 82%. LA conduit function was impaired in 80% of patients.

#### 3.5.2. Bivariate Analysis

The results of the bivariate analysis with demographic, echocardiographic, and therapeutic parameters are shown in Tables 3 and 4. The LA parameters are significant. The LA maximal volume was increased in non-diabetic patients (*p* = 0.037). The reservoir function was impaired in patients who take beta-blockers compared with other antihypertensive drugs (*p* = 0.023). The conduit function was impaired in obese patients (*p* = 0.015). However, the LA pump function was significantly impaired in patients treated with calcium antagonists compared with other antihypertensive drugs (*p* = 0.012).

The results of analysis of the heart rate with antihypertensive drugs demonstrate that the average of the heart rate in patients taking calcium antagonists was significantly different. However, no significant difference was observed in patients taking beta-blockers.

## 4. Discussion

AHT is a global public health problem. It contributes to the burden of disease through heart disease, stroke, and kidney failure. The LA is a structure whose importance is known for a long time but whose exploration was unusual in echocardiography until now. In recent years, however, according with the cardiology literature, several articles on LA size and function have been explored, using 2D, 3D, and Doppler ultrasound imaging techniques, but also through the study of myocardial deformation (Strain). Studies of LA function related to hypertension have not been done before in Morocco. The only one was done in Tunisia in 2015 [14] on the same subject on the evaluation of the LA in 50 hypertensive patients, found values close to ours which are, for the total, passive, and active fractions, respectively, 50%, 22%, and 35%. An American study done in 2014 by Miyoshi et al. [15] on the evaluation of the relationship between LA reservoir function and diastolic dysfunction in hypertensive patients, by two-dimensional echocardiography and speckle tracking imaging, found LA reservoir fraction at 51.9% in hypertensive patients with LAVmax>29 ml/m^2^ and at 49,9% in patients with LAVmax <29 ml/m^2^. Apart the hypertension, the impairment of LA function has been demonstrated by other studies in other pathological situations such as overload disease (Fabry) and atrial fibrillation [16, 17]. In our series, the LA reservoir function judged on the total emptying fraction was more important. The conduit function is impaired in hypertensive patients with a significantly lower passive emptying fraction (24.6 ± 10%); this decrease is greater in patients with left ventricular hypertrophy and diabetes. The LA pump function is increased in hypertensive patients with an elevated active emptying fraction (39.5 ± 15%); this increase is more marked in the presence of diastolic dysfunction and patients treated by calcium antagonists. These results are similar in Tunisia study, which showed that the LA conduit function is impaired in patients with LVH (*p* = 0.02) compared to the control, while the pump function is increased in the presence of dysfunction diastolic (*p* = 0.029), and also to that of the United States who have found that diastolic dysfunction was factor on increasing in the reservoir function and consequently the pump function. There is also a very strong association between all the parameters of the LA (LA volumes and function) and the filling pressures. These convincing results are those of Murata et al. [18], who demonstrated in 2008, through 106 patients with diastolic dysfunction, that the LA maximum and minimum volumes and the LA total emptying volume were directly correlated with LV filling pressures. The results obtained in our series are consistent with these findings. Antihypertensive treatment also changes the LA function; an impairment of reservoir function is observed in patients treated with beta-blockers (BB). These results follow those found by Sardana et al. [19] on the assessment of the impact of BB on LA function in hypertensive patients. Potential mechanisms linking BB use with atrial dysfunction may include any of the following factors, alone or in combination: direct negative inotropic effects of BB on the LA myocardium; LA dysfunction secondary to negative inotropic or lusitropic effects of BB on the LV; worsening LA-LV-aortic coupling due to the effects of BB on central hemodynamics and decreasing of LVEF in patient using BB, the association between LA function and BB use persisted after adjustment for LVEF. Previous studies indicate that central pulsatile hemodynamics is associated with LA remodeling and dysfunction in hypertension [19]. The CAFE study [20] recruited 2199 patients in 5 Anglo-Scandinavian Cardiac Outcomes Trial (ASCOT) centers demonstrating that BP-lowering drugs (atenolol ± thiazide versus amlodipine ± perindopril) can have substantially different effects on central aortic pressures and hemodynamics despite a similar impact on brachial BP. There were substantial reductions in central aortic pressures with the amlodipine regimen. This effect appears to be associated essentially with the negative inotropic effect of the BB which, delaying the occurrence of the peak systolic ejection, increases the probability of a summation of the pressure wave with the reflected pulse wave increasing the systolic pressure in the aorta. Central aortic pulse pressure may be a determinant of clinical outcomes, and differences in central aortic pressures may be a potential mechanism to explain the different clinical outcomes between the 2 BP treatment arms in ASCOT. In the LIFE trial [21], higher pulse pressure was observed in the atenolol-based group when compared to the losartan-based group, and this independently predicted increased risk of cardiovascular morbidity and mortality in these participants. However, we have not found a study in the literature which demonstrates that calcium antagonists impaired the LA pump function, such in this study. In Takami study [22], LVH and diastolic function improved in the cilnidipine and amlodipine groups, but not in the nifedipine group; the LV mass index had significantly decreased when it was evaluated 3 months after the initiation of treatment in the cilnidipine group and when it was evaluated 6 months after the initiation of treatment in the amlodipine group with a significant increase in the E/A ratio were observed after 3 months of treatment in the cilnidipine and amlodipine groups but not in the nifedipine group. The improvement of LVH and diastolic function with calcium antagonists appears to be associated with the suppression of sympathetic nerve activity by the blockade of N-type calcium channels. Other imaging modes, 3D imaging represents one of the latest novelties that bring very significant information in the study of the LA volumes and function. Rohner et al. [23], in their study, stated that the LA volumes and function made by the 3D ETT and the 2D could have the same overall results. Recently, the study conducted by Mochizuki et al. showed that the study by three-dimensional echocardiography with a strain had a good reproducibility of the left atrial function in a good number of pathologies (AHT, CMD, and amylose) [24].

## 5. Conclusion

This study demonstrates that AHT induced LA dilatation, increased pump and reservoir function, and impaired conduit function; these impacts appear to be related to LVH and the degree of LV diastolic dysfunction. The most powerful factors for this repercussion are diabetes, obesity, and antihypertensive drugs. Further investigations are sensible to clarify this study: three-dimensional echocardiography, speckle tracking for strain-rate.

## Figures and Tables

**Table 1:** Demographic and clinical variables in 100 subjects of the population's sample in which all data were available.

Qualitative variables	*N* = 100	Proportion (%)
Men	58	58
Diabetic	44	44
Obese	46	46
Smoking	17	17
Menopause	08	19
Sedentary	43	43
Grade AHT II	62	62
Asymptomatic	61	61
Stroke	03	03
Stable angina	06	06
LVH on ECG	26	26
No added salt diet	05	05
CA	20	20
ACE	39	39
ARAII	34	34
BB	19	19
DT	24	24
Statin	17	17
Aspirin	12	12
Monotherapy	47	47
Dual therapy	40	40
Triple therapy	13	13

Quantitative variables	Means	

Age (years)	57,3 (41–79)	
BMI (kg/m^2^)	27,2 (21–36)	
Hypertension duration (years)	5,7 (1–8)	
Systolic blood pressure (mmhg)	12,72 (105–162)	
Diastolic blood pressure (mmhg)	78,3 (63–102)	
Heart rate (bpm)	63,2 (51–86)	

BMI: body mass index; CA: calcium antagonists; ACE: angiotensin-converting enzyme; ARAII: angiotensin II receptor antagonists; BB: beta-blockers; DT: diuretics; LVH: left ventricular hypertrophy; ECG: electrocardiogram.

**Table 2:** Echocardiographic variables.

Left ventricular parameters	

Quantitative variables	Means

LVEF (%)	65 ± 4
LVEDD (mm)	49 ± 7
LVESD (mm)	30, 2 ± 7
LVM (g/m^2^)	116,7 ± 19

Qualitative variables	*N* = 100

Diastolic dysfunction I (*n*)	56
Diastolic dysfunction II (*n*)	29
Diastolic dysfunction III (*n*)	01
Diastolic dysfunction undetermined (*n*)	14
LVH	65

Left atrial parameters	

Quantitative variables	Means

Anteroposterior diameter (mm)	50, 5 ± 8
LA area (cm^2^)	17, 6 ± 3
LAVmax/BSA (ml/m^2^)	29 ± 7
LAVmin/BSA (ml/m^2^)	11 ± 4
LAVpre-A/BSA (ml/m^2^)	18, 5 ± 5
LAtotEF (%)	54, 9 ± 12
LAVpassEF (%)	24, 6 ± 10
LAVactifEF (%)	39, 5 ± 15
Expansion index	1.44

Qualitative variables	*N* = 100

Increased reservoir function (*n*)	85
Decreased conduit function (*n*)	80
Increased pomp function (*n*)	82

LVEF: left ventricular ejection fraction; LVM: left ventricular mass; LVH: left ventricular hypertrophy; LA: left atrial; LAVmax: maximal left atrial volume; BSA: body surface area; LAVmin: minimal left atrial volume; LAVpré-A: left atrial volume before atrial contraction; totEF: total emptying fraction; passEF: passive emptying fraction; actifEF: active emptying fraction; LVEDD: left ventricular end-diastolic dimension.

**Table 3:** Variation of LA maximal volume according to demographic and clinical parameters.

Variables		LA maximal volume increased	
		Effective (percentage)	*p* value
Age group	40-49	01 (11,1%)	N/A
	50–59	06 (36,7%)	
	60-69	01 (11,1%)	
	70-79	01 (11,1%)	

Genre	Men	04 (44,4%)	0,3
	Women	05 (55,6%)	

Grade AHT	I	0 (0%)	N/A
	II	06 (66,7%)	
	III	03 (33,3%)	

Diabetes	Yes	01 (11,1%)	**0,037**
	No	08 (88,9%)	

Dyslipidemia	YES	01 (11,1%)	0,36
	No	08 (88,9%)	

Sedentarite	Yes	03 (33,3%)	0,4
	No	06 (66,6%)	

Obesity	Yes	03 (33,3%)	0,33
	No	06 (66,6%)	

Smoking	Yes	01 (11,1%)	0,52
	No	08 (88,9%)	

Coronary heredity	Yes	01 (11,1%)	0,24
	No	08 (88,9%)	

Menopause	Yes	01 (11,1%)	0,54
	No	08 (88,9%)	

CA	Yes	01 (11,1%)	0,42
	No	08 (88,9%)	

ACE	Yes	02 (22,2%)	0,29
	No	07 (77,8%)	

ARAII	Yes	01 (11,1%)	0,58
	No	08 (88,9%)	

BB	Yes	00 (0%)	0,61
	No	09 (100%)	

Diastolic dysfunction	I	04 (44,44%)	N/A
	II	01 (11,11%)	
	III	04 (44,44%)	
	Undetermined	0 (0%)	

LVH	Yes	07 (77,8%)	N/A
	No	02 (22,2%)	

CA: calcium antagonists; ACE: angiotensin-converting enzyme; ARAII: angiotensin II receptor antagonists; BB: beta-blockers; N/A: not applicable; LVH: left ventricular hypertrophy.

**Table 4:** Bivariate analysis of LA function.

Variable**s**		LA reservoir impaired		LA conduit impaired		LA pomp impaired	
		Effective (%)	*p* value	Effective (%)	*p* value	Effective (%)	*p* value
Age group	40-49	02 (13,33%)	0,94	08 (10%)	N/A	05 (27,77%)	N/A
	50-59	07 (46,66%)		42 (52,5%)		10 (55,55%)	
	60-69	05 (33,33%)		22 (27,5%)		02 (11,11%)	
	70-79	01 (6,66%)		08 (10%)		01 (5,55%)	

Genre	Men	09 (60%)	0,54	50 (62,5%)	0,59	12 (66,66%)	0,29
	Women	06 (40%)		30 (37,5%)		06 (33,33%)	

Grade AHT	I	00 (0%)	0,79	03 (3,8%)	N/A	00 (0%)	NA
	II	11 (73,33%)		50 (62,5%)		12 (66,66%)	
	III	04 (26,66%)		27 (33,7%)		06 (33,33%)	

Diabetes	Yes	08 (53,33%)	0,3	32 (30%)	0,08	09 (50%)	0,37
	No	07 (46,66%)		48 (60%)		09 (50%)	

Dyslipidemia	Yes	03 (20%)	0,5	15 (18,8%)	0,1	06 (33,33%)	0,16
	No	12 (80%)		65(81,2%)		12 (66,66%)	

Sedentarite	Yes	07 (46,66%)	0,48	32 (30%)	0,16	08 (44,44%)	0,54
	No	08 (53,33%)		48 (60%)		10 (55,55%)	

Obesity	Yes	06 (40%)	0,41	32 (30%)	**0,015**	10 (55,55%)	0,26
	No	09 (60%)		48 (60%)		08 (44,44%)	

Smoking	Yes	04 (26,66%)	0,23	14 (17,5%)	0,54	04 (22,22%)	0,36
	No	11 (73,33%)		66 (82,5%)		14 (77,77%)	

Coronary heredity	Yes	00 (0%)	0,61	03 (3,8%)	0,5	01 (5,55%)	0,45
	No	15 (100%)		77 (96,2%)		17 (94,44%)	

Menopause	Yes	03 (20%)	0,09	06 (7,5%)	0,5	03 (16,66%)	0,15
	No	12 (80%)		74 (92,5%)		15 (83,33%)	

CA	Yes	01 (6,66%)	0,14	17 (21,25%)	0,39	00 (0%)	**0,012**
	No	14 (93,33%)		63 (78,75%)		18 (100%)	

ACE	Yes	02 (13,33%)	0,57	08 (10%)	0,19	06 (33,33%)	0,07
	No	13 (86,66%)		72 (90%)		12 (66,66%)	

ARAII	Yes	01 (6,66%)	0,59	06 (7,5%)	0,25	01 (5,55%)	0,49
	No	14 (93,33%)		74 (92,5%)		17 (94,44%)	

BB	Yes	03 (20%)	**0,023**	05 (6,25%)	0,31	01 (5,55%)	0,63
	No	12 (80%)		75 (93,75%)		17 (94,44%)	

Diastolic dysfunction	I	10 (66,6%)	0,4	46 (57,5%)	N/A	07 (38,88%)	N/A
	Ii	02 (13,33%)		23 (28,75%)		04 (22,22%)	
	Iii	00 (0%)		01 (1,25%)		01 (5,55%)	
	Undetermined	03 (20%)		10 (12,5%)		06 (33,33%)	

LVH	Yes	12 (80%)	0,15	52 (65%)	0,6	14 (77,77%)	0,16
	No	03 (20%)		28 (35%)		04 (22,22%)	

CA: calcium antagonists; ACE: angiotensin-converting enzyme; ARAII: angiotensin II receptor antagonists; BB: beta-blockers; N/A: not applicable; LVH: left ventricular hypertrophy.

## Data Availability

The data used to support the findings of this study are available from the corresponding author upon request.
